# Prevalence of and risk factors for tuberculosis among healthcare workers in Chinese tuberculosis facilities

**DOI:** 10.1186/s40249-018-0407-6

**Published:** 2018-03-24

**Authors:** Xiao-Ning Wang, Tian-Lun He, Meng-Jie Geng, Yu-Dan Song, Ji-Chun Wang, Min Liu, Sarah Jayne Hoosdally, Ana Luíza Gibertoni Cruz, Fei Zhao, Yu Pang, Yan-Lin Zhao, Guang-Xue He

**Affiliations:** 1 Department of Science and Technology, Chinese Center for Disease Control and Prevention (China CDC), Beijing, China; 2 Chaoyang District Center for Disease Control and Prevention, Beijing, China; 3 School of Economics and Management, Beijing University of Technology, Beijing, China; 4 Department of Infectious Disease Control and Prevention, China CDC, Beijing, China; 5 National Center for Chronic Disease Control and Prevention, China CDC, Beijing, China; 6 Nuffield Department of Medicine, University of Oxford, John Radcliffe Hospital, Oxford, UK; 7 National Center for Tuberculosis Control and Prevention, China CDC, Beijing, China

**Keywords:** Tuberculosis, Prevalence, Risk factors, Healthcare workers, China

## Abstract

**Background:**

China is one of 22 countries with a high tuberculosis (TB) burden in the world. Healthcare workers (HCWs) have a high risk of contracting *Mycobacterium tuberculosis* infection due to insufficient infection control practices. We conducted a cross-sectional study to explore the prevalence of TB and its associated risk factors among HCWs in Chinese TB facilities.

**Methods:**

Two hundred and forty-one TB facilities employing a total of 9663 HCWs were selected from 12 provinces in China to represent healthcare settings at the provincial, prefectural, and county levels. Structured questionnaires were used to collect information on TB infection control practices and HCWs in those facilities. Data was double entered into EpiData 3.1; TB prevalence and associated risk factors were analyzed using SPSS 21.0 with bivariate and multivariate regression models.

**Results:**

The results showed that 71 HCWs had been diagnosed with TB, accounting for a prevalence of 760/100 000. The multivariate analysis showed that associated risk factors included belonging to the age group of 51 years and above (a*OR*: 6.17, 95% *CI*: 1.35–28.28), being a nurse (a*OR* = 3.09, 95% *CI*: 1.15–8.32), implementation of 0–9 items of management measures (a*OR* = 2.57, 95% *CI*: 1.37–4.80), and implementation of 0–1 items of ventilation measures (a*OR* = 2.42, 95% *CI*: 1.31–4.47).

**Conclusion:**

This was the first national large sampling survey on TB prevalence among HCWs in China. It was found that the implementation of TB infection control practices in some facilities was poor. The TB prevalence in HCWs was higher than that in the general population. Therefore, TB infection control practices in Chinese medical facilities should be strengthened.

**Electronic supplementary material:**

The online version of this article (10.1186/s40249-018-0407-6) contains supplementary material, which is available to authorized users.

## Multilingual abstract

Please see Additional file 1 for translations of the abstract into the five official working languages of the United Nations.

## Background

Tuberculosis (TB) is caused by the bacillus *Mycobacterium tuberculosis* (MTB) and remains one of the major problems threatening public health worldwide [1]. There were 10.4 million new cases diagnosed in 2015, accounting for a global estimated incidence of 142/100 000 [1].

China is one of 22 high TB burden countries for both TB and multidrug resistant TB cases [1–4]. The fifth national TB epidemiological survey conducted in 2010 showed that national prevalence rates of active TB, sputum smear-positive, and sputum culture-positive cases were 459/100 000, 66/100 000, and 119/100 000, respectively [5]. Hence, the epidemic of TB is one of the most important public health problems that China currently faces.

Tuberculosis is primarily a respiratory infectious disease; infection is airborne and transmitted via aerosols. Healthcare workers (HCWs) that are exposed to MTB have a 10- to 20-fold risk of contracting TB than the general population [6–8]. The paucity of occupational health programs tailored to HCWs resulting in inadequate information and equipment to prevent or minimize exposure to TB, in addition to structurally obsolete healthcare facilities with inadequate ventilation, are likely key drivers of such increased risks. Moreover, as people with latent TB infections (LTBIs) usually go undiagnosed, the implementation of TB infection control policies in healthcare facilities, which includes the diagnosis of LTBIs, is imperative to decrease TB transmission, incidence, and prevalence among HCWs [9]. Systematic application of TB infection risk assessments, formulation and implementation of infection control strategies, early diagnosis, and prompt treatment are vital strategies that should be incorporated into TB infection control programs to effectively reduce nosocomial TB infection risk [7].

Despite the World Health Organization establishing an international policy that aims to protect HCWs from acquiring TB [8], infection control practices have been insufficiently implemented in China, where LTBI screening policy, personal protective strategies, and risk assessments that should be embedded in routine workflows [9, 10] are not consistently undertaken. It is therefore imperative to understand the current status of TB infection control in Chinese TB facilities, in which HCWs are at a higher risk of contracting TB infection.

Study conducted in 22 TB facilities in three Chinese provinces provided a general understanding on the implementation of TB infection control policies and associated risk factors for TB infection in HCWs [11, 12]. This study, however, aims to assess the nationwide status of TB infection control in China: we conducted a cross-sectional study involving TB healthcare facilities of different levels of complexity in 12 Chinese provinces. We aimed to determine the prevalence of TB among HCWs, identify the key components driving TB infection risk among HCWs, and lay out the evidence for policy development in TB infection control.

## Methods

### Study design and sampling

Based on the composition of HCWs at the different facilities, stage of the implementation of TB infection control measures, and willingness to be involved in this study, healthcare facilities from four eastern provinces (Jiangsu, Zhejiang, Shandong, and Liaoning); four central provinces (Anhui, Jiangxi, Henan, and Shanxi); and four western provinces (Shaanxi, Yunnan, Gansu, and Sichuan) were enrolled in the study. In each province, facilities from three prefectures were included and from each prefecture, facilities from four counties were included.

In order to reflect the organizational and structural heterogeneities of healthcare facilities in China, the TB facilities were selected as follows: (i) one TB dispensary and one TB specialized hospital from each provincial level; (ii) one TB dispensary or one infectious disease hospital from each prefectural level; (iii) one specialized hospital or general hospital with a TB ward from each prefectural level and; (iv) one TB dispensary with an outpatient department or designated hospital with an outpatient department, or general hospital with a TB ward from each county level. In total, 241 facilities were enrolled in this study.

All the HCWs in these facilities including administrative staff, doctors, nurses, and medical technicians were enrolled. Both long-term and temporary employees were included. In total, 9663 HCWs participated in this study.

### Data collection

Data were collected via structured questionnaires between February and October 2013.

Provincial-level staff members were trained on the principles and practices of TB infection control by researchers from the Department of Science and Technology at the Chinese Centre for Disease Control and Prevention (China CDC). Those working in provincial facilities were responsible for collecting the data in all facilities (“investigators”) following training and assessment by the China CDC. The structured questionnaires were designed in accordance with the Chinese standard practice procedures for TB infection control [13] and included TB infection control questions around healthcare facilities and HCWs.

The questionnaire for healthcare facilities contained 11 sections (see Table 1) and was completed by the investigators. It elicited general background information on the facility, its management structure, administrative implementation, and the implementation of infection control strategies in outpatient departments, laboratories, or inpatient wards. Observations were made about administrative measures, design of the premises, environmental measures, and individual protection of HCWs in outpatient departments, wards, and laboratories.

**Table 1:** The definition for variables of implementation of infection control (line 126 in page 6)

Number	Variables	Definition
a	with in-patient wards	whether the facility included in-patient wards for TB patients
b	screening for patients	whether patients were screened for TB
c	department and staff setting	department and staff composition: whether there was a dedicated department for TB infection prevention and control; specialist and sufficient staff
d	infection control policies and budgets	infection control policies and budgets: whether a program for TB infection control existed; existing an established referral system; existing appropriate and dedicated funding
e	staff training Implementation	whether there was pre-employment training program in TB infection prevention and control, provision of continuous education; provision of review courses and activities
f	infection control evaluation implementation	details of incorporation of TB infection control practices to performance indicator systems and evaluation of the environmental implementation for TB infection control
g	administrative implementation	whether dedicated area for coughing patients are provided during appointments; appropriately designed sputum collection area according to building standards; provision of facial tissues or medical masks for patients; measurement of time spent by patients at health care facilities (with an aim to shorten length of stay); firstly receiving samples of suspected infectious TB cases in laboratories; firstly treating suspected infectious TB cases in in-patient wards; with details of any isolation system; separating patients with and without a cough; separating TB cases (infectious or multi-drug resistant disease) from non-TB patients in in-patient wards or out-patient departments
h	promotion and education implementation	whether promotional materials about TB infection control is offered in out-patient departments and wards, including cough etiquette and respiratory hygiene in out-patient departments and wards, ensuring patients keep good cough etiquette and wear surgical masks in wards;
i	individual protection	carrying out TB infection control education, conducting health check-up examinations on an yearly basis for HCW; wearing N95 medical masks when collecting smear; wearing N95 medical masks among HCWs in out-patient wards or laboratories; conducting fitting tests of N95 medical masks regularly;
j	floor plan design	the designation rationality in the waiting area, or in out-patient departments, or in laboratories, or in-patient wards;
k	ventilation	good ventilation in out-patient departments; HCWs in out-patient departments sitting upwind of patients; installation of ventilation equipment in out-patient departments or wards;
l	ultraviolet germicidal lamps installation and maintenance	installing ultraviolet germicidal lamps in out-patient departments, or wards or laboratories; having a good maintenance plan in out-patient departments, or wards or laboratories.

The questionnaire for the HCWs elicited information on demographics, duration of employment at TB facility, professional category and length of service in current position, history of training to conduct TB infection control, smoking history, previous and/or current history of TB infection, and, if infected, details of laboratory results and treatment. The investigators collated this information and completed the questionnaires by reviewing professional and medical records, and by interviewing the relevant staff members in the event clarification was required.

### Quality control

Data were collected by investigators who were deemed eligible after being examined by China CDC researchers. All healthcare facilities that were approached by our team agreed to participate in the study. Senior researchers from the China CDC were available to provide technical support and supervision as required. All questionnaires were double-checked for completeness by investigators. Healthcare facilities in Shandong, Henan, and Shanxi provinces in the eastern, central, and western provinces, respectively, were randomly selected for audit of filled questionnaires by senior researchers.

Data were double entered into EpiData 3.1(manufacture: The EpiData Association, location: Denmark) and checked for coincidence rate, abnormal values, and logical errors by trained data entry clerks.

### Data analysis

Data were analyzed using SPSS 21.0 (manufacture: Chinese SPSS company, serial number: 20131105-LSZU, location: Shanghai in China). The associations between the prevalence of TB and its risk factors were assessed by bivariate regression analysis. In the bivariate analysis, variables (*P* ≤ 0.2) were retained in the multivariate regression analysis. Then, forward selection of variables was used in the multivariate regression model, and odds ratios (*OR*s) and their 95% confidence intervals (*CI*s) were determined.

## Results

### Distribution of survey sites enrolled in the study

Of the 241 facilities, 29 were excluded from the study as they did not have outpatient departments or wards, or lacked information on managerial strategies and environmental control to tackle nosocomial TB infections (e.g. floor plans, ventilation systems, ultraviolet germicidal lamps, etc.). Therefore, the final analysis was undertaken using data provided by 212 facilities and 9336 HCWs.

Of the 212 survey sites, 30.7% were in eastern China, 34.0% were in central China, and 35.3% were in western China. To break it down further, 71.7, 22.2, and 6.1% of the sites were at the county, prefectural, and provincial levels, respectively. Of all the sites, 78.8% were TB dispensaries and only 3.3% were TB specialized hospitals; 46.2% had wards, outpatient departments, and laboratories; and 92.0% had TB prevention departments (see Table 2).

**Table 2:** Characteristics of survey sites enrolled in this study (line 175 in page 8)

	Facilities			
Characteristics	Total N (%)	Eastern n (%)	Middle n (%)	Western n (%)
Total	212	65	72	75
Unit level				
Province	13 (6.1)	3 (1.4)	4 (1.9)	6 (2.8)
Prefecture	47 (22.2)	15 (7.1)	15 (7.1)	17 (8.0)
County	152 (71.7)	47 (22.2)	53 (25.0)	52 (24.5)
Facility type^a^				
TB dispensary	170 (80.2)	46 (21.7)	63 (29.7)	61 (28.8)
TB specialized hospital	7 (3.3)	2 (0.9)	1 (0.5)	4 (1.9)
Infectious disease hospital with TB department	13 (6.1)	6 (2.8)	6 (2.8)	1 (0.5)
General hospital	40 (18.9)	23 (10.8)	5 (2.4)	12 (5.7)
Facility category				
With out-patient department and laboratory	114 (53.8)	16 (7.5)	49 (23.1)	49 (23.1)
With in-patient ward, out-patient department and laboratory	98 (46.2)	49 (23.1)	23 (10.8)	26 (12.3)
Department setting^b^				
With department of TB prevention	188 (88.7)	54 (25.5)	69 (32.5)	65 (30.7)
With department of respiration medicine	53 (25.0)	28 (13.2)	15 (7.1)	10 (4.7)
With department of infection control	71 (33.5)	36 (17.0)	17 (8.0)	18 (8.5)

Note: ^a^3 of specialized hospitals With TB department and 15 of general hospitals also were TB dispensaries

^b^30 facilities had the departments of TB prevention, respiration medicine and infection control; 7 facilities had two departments of TB prevention and respiration medicine; 17 facilities had two departments of TB prevention and infection control; 16 facilities had two departments of respiration medicine and infection control

Of the 9336 HCWs, 49.0% were from the eastern, 23.3% from the central, and 27.7% from the western provinces, respectively. Of all the HCWs, 54.5% worked in prefectural facilities. In terms of sex, 35.2% were male and 64.8% were female. The average age of HCWs was 38 years old (range 18–81) and 31.8% were below 30 years of age. In terms of education, 39.9% had a bachelor’s degree, 31.1% had a junior college degree, and only 4.7% had a master’s degree. The average duration of employment in all facilities was 16.7 years (range 1–64 years); 25.4% worked in TB prevention facilities for less than 5 years and 26.1% worked for more than 26 years. In terms of qualifications, 33.4% had a primary professional qualification and 11.4% had a senior professional qualification. Of all the HCWs, 35.1% were doctors and 28.4% were nurses (see Table 3).

**Table 3:** The demographic information of HCWs (line 184 in page 9)

Demographic information	Total N (%)	Province n (%)	Prefecture n (%)	County n (%)
Gender				
Male	3289 (35.2)	648 (6.9)	1609 (17.2)	1032 (11.1)
Female	6047 (64.8)	1333 (14.3)	3479 (37.3)	1235 (13.2)
Age (years)				
≤ 30	2971 (31.8)	792 (8.5)	1671 (17.9)	508 (5.4)
31–40	2436 (26.1)	438 (4.7)	1320 (14.1)	678 (7.3)
41–50	2740 (29.4)	510 (5.5)	1487 (15.9)	743 (8.0)
≥ 51	1189 (12.7)	241 (2.6)	610 (6.5)	338 (3.6)
Education				
Master	438 (4.7)	203 (2.2)	216 (2.3)	19 (0.2)
bachelor	3726 (39.9)	808 (8.6)	2231 (23.9)	687 (7.4)
junior college	2900 (31.1)	565 (6.1)	1500 (16.1)	835 (8.9)
Senior school and below	2272 (24.3)	405 (4.3)	1141 (12.2)	726 (7.8)
Profession title^a^				
senior	1064 (11.4)	252 (2.7)	571 (6.1)	241 (2.6)
intermediate	2627 (28.1)	498 (5.3)	1466 (15.7)	663 (7.1)
primary	3120 (33.4)	730 (7.8)	1615 (17.3)	775 (8.3)
Other	2525 (27.1)	501 (5.4)	1436 (15.4)	588 (6.3)
Occupation				
administrative	2262 (24.2)	435 (4.7)	1258 (13.5)	569 (6.1)
Nurse	2648 (28.4)	656 (7.0)	1737 (18.6)	255 (2.7)
doctor	3276 (35.1)	813 (8.7)	1586 (17.0)	877 (9.4)
Medical technician	1150 (12.3)	77 (0.8)	507 (5.4)	566 (6.1)
Duration of employment (years)				
≤ 5	2369 (25.4)	541 (5.8)	1381 (14.8)	447 (4.8)
6–15	2113 (22.6)	477 (5.1)	1170 (12.5)	466 (5.0)
16–25	2418 (25.9)	432 (4.6)	1281 (13.7)	705 (7.6)
≥ 26	2430 (26.1)	531 (5.7)	1256 (13.5)	643 (6.9)

^**a**^senior: the professional title of HCWs who had passed the hardest professional examination, and had more work experience, more medical knowledge; intermediate: the professional title of HCWs who had passed harder professional examination, and worked several years and had some medical knowledge; primary: the professional title of HCWs who had got the doctor license; other: none title

Regarding infection with TB, 71 HCWs had been diagnosed with TB during the study period. This comprised: 30 nurses (42.3%), 28 doctors (39.4%), eight administrative staff members (11.3%), and five medical technicians (7.0%). Of the 71 TB cases, 28 (39.4%) were male and 43 (60.6%) were female; 28 (39.4%) were 30 years old or less. Five (7.0%) of the 71 TB cases were classified as sputum smear-positive and two (2.8%) were sputum-culture positive. In contrast, three (4.2%) had no bacterial evidence. In terms of outcome, 50 (81.7%) had been cured, while 13 (18.3%) were still under treatment upon the survey’s completion (see Table 4).

**Table 4:** Characteristics of TB patients among HCWs (line 193 in page 9)

Characteristics of TB patients	n	New case	Relapse case
Bacterial diagnosis			
SS+	5	2	3
SS-	61	36	25
C+	2	0	2
non sputum smear	3	1	2
Treatment outcome			
Cured	58	27	31
Under treatment	13	12	1

*SS+ sputum smear positive, *SS-* sputum smear negative, *C+* culture positive

Of all the facilities under study, 85.4% (181/212) had carried out suspected symptom screening for healthcare visitors. Moreover, 92.3% (12/13) of provincial facilities successfully implemented this practice. Of all the facilities, 99.1% (210/212) had implemented TB infection control evaluation, but only one measure was evaluated in 66.0% (140/212) of the facilities. Of the facilities, 39.6% (84/212) conducted administrative implementation, with 17.0% (36/212) conducting 1–9 administrative measures. Almost all facilities (97.2%; 206/212) had appropriate ventilation systems and 94.3% (200/212) implemented 2–4 measures. In addition, 36.3% (77/212) conducted implementation of ultraviolet germicidal lamps installation with proper maintenance (see Table 5).

**Table 5:** Infection control implementation conducted in different level facilities (line 203 in page 9)

Implementation of infection control	Total n (%)	Province n (%)	Prefecture n (%)	County n (%)
With in-patient wards^a^	98 (46.2)	10 (4.7)	40 (18.9)	48 (22.6)
screening for patients^b^	181 (85.4)	12 (5.7)	36 (17.0)	133 (62.7)
Items of department and staff setting^c^				
0–1	39 (18.4)	3 (1.4)	8 (3.8)	28 (13.2)
2	103 (48.6)	9 (4.2)	32 (15.1)	62 (29.2)
Items of infection control policies and budgets^d^				
0-1	37 (17.5)	2 (0.9)	6 (2.8)	29 (13.7)
2–3	167 (78.8)	11 (5.2)	38 (17.9)	118 (55.7)
Items of staff training Implementation^e^				
0–2	76 (35.8)	3 (1.4)	9 (4.2)	64 (30.2)
3	134 (63.2)	10 (4.7)	38 (17.9)	86 (40.6)
Items of infection control evaluation implementation^f^				
0-1	140 (66.0)	6 (2.8)	14 (6.6)	120 (56.6)
2	70 (33.0)	6 (2.8)	33 (15.6)	31 (14.6)
Items of administrative implementation ^g^				
0–9	36 (17.0)	5 (2.4)	15 (7.1)	16 (7.5)
10-12	48 (22.6)	3 (1.4)	20 (9.4)	25 (11.8)
Items of promotion and education implementation^h^				
0–3	3 (1.4)	0 (0.0)	2 (0.9)	1 (0.5)
4–6	85 (40.1)	10 (4.7)	36 (17.0)	39 (18.4)
Items of individual protection^i^				
0–5	138 (65.1)	9 (4.2)	29 (13.7)	100 (47.2)
6	26 (12.3)	3 (1.4)	7 (3.3)	16 (7.5)
Items of floor plan design^j^				
0–2	20 (9.4)	4 (1.9)	8 (3.8)	8 (3.8)
3–4	67 (31.6)	5 (2.4)	28 (13.2)	34 (16.0)
Items of ventilation^k^				
0–1	6 (2.8)	0 (0.0)	2 (0.9)	4 (1.9)
2–4	200 (94.3)	13 (6.1)	41 (19.3)	146 (68.9)
Items of ultraviolet germicidal lamps installation and maintenance^l^				
0–4	25 (11.8)	3 (1.4)	13 (6.1)	9 (4.2)
5–6	52 (24.5)	6 (2.8)	19 (9.0)	27 (12.7)

### Prevalence of and risk factors for TB

The overall prevalence of TB among HCWs was 760/100 000 (71/9336 × 100 000). The TB prevalence of males was 851/100 000 and of females it was 711/100 000. The TB prevalence of non-smokers was 756/100 000 and of smokers it was 973/100 000. No statistical significance (*P* > 0.05) was found between educational status (master’s degree: 913/100 000; bachelor’s degree: 724/100 000; junior college: 931/100 000; and junior school or below: 572/100 000).

Based on the result of the bivariate analysis, variables such as inpatient wards, screening of patients, department and staff setting, staff training, and evaluation measures showed no association with TB prevalence. Tuberculosis prevalence was associated with variables related to the healthcare setting, such as different level of healthcare facility, geographical area, local implementation of policies and use of allocated budgets, availability of personal protective equipment, implementation of administrative measures, promotion and education, floor plan design, ventilation, ultraviolet lamp installation and maintenance, and variables related to the individual HCWs, such as age group, occupational category, professional qualification, and duration of employment. All these variables were found to have a *P*-value of ≤0.2 and were entered in the multivariate regression model (see Table 6).

**Table 6:** Risk factors of TB prevalence among HCWs (line 221 in page 10 and 227 in page 11)

Variables	Total *n*	Prevalence n (1/100000)	Crude *OR* (95% CI)	*P*	Adjusted *OR* (95% CI)
Facility level					
provincial	1981	10 (505)	1		1
prefectural	5088	42 (825)	1.64 (0.82–3.28)	0.161	1.61 (0.69–3.76)
county	2267	19 (838)	1.67 (0.77–3.59)	0.193	1.90 (0.73–4.98)
Region					
eastern	4574	28 (612)	1		1
middle	2176	20 (919)	1.51 (0.85–2.68)	0.164	1.18 (0.59–2.34)
western	2586	23 (889)	1.46 (0.84–2.54)	0.183	0.75 (0.38–1.47)
Gender					
male	3289	28 (851)	1		–
female	6223	43 (711)	0.83 (0.52–1.35)	0.457	–
Age (years)					
≤ 30	2971	28 (942)	1		1
31–40	2436	19 (779)	0.83 (0.46–1.48)	0.523	1.07 (0.47–2.40)
41–50	2740	12 (438)	0.46 (0.24–0.91)	0.026	1.08 (0.33–3.51)
≥ 51	1189	12 (1009)	1.07 (0.54–2.11)	0.842	6.17 (1.35–28.28)
Education					
master	438	4 (913)	1		–
bachelor	3726	27 (724)	0.79 (0.28–2.27)	0.665	–
junior college	2900	27 (931)	1.02 (0.36–2.93)	0.971	–
junior school and below	2272	13 (572)	0.62 (0.20–1.92)	0.412	–
Profession qualification					
senior	1064	11 (1034)	1		1
intermediate	2627	19 (723)	0.70 (0.33–1.47)	0.344	0.60 (0.27–1.31)
primary	3120	32 (1026)	0.99 (0.50–1.98)	0.982	0.79 (0.33–1.87)
other	2525	9 (356)	0.34 (0.14–0.83)	0.017	0.40 (0.10–1.63)
Occupation					
Medical technician	1150	5 (435)	1		1
administrative	2262	8 (354)	0.81 (0.27–2.49)	0.717	1.56 (0.31–7.74)
nurse	2648	30 (1133)	1.97 (0.76–5.13)	0.046	3.09 (1.15–8.32)
doctor	3276	28 (855)	2.62 (1.02–6.78)	0.162	2.21 (0.83–5.86)
Duration of employment (years)					
≤ 5	2369	24 (1013)	1		1
6–15	2113	18 (852)	0.84 (0.45–1.55)	0.577	0.97 (0.45–2.09)
16–25	2418	16 (662)	0.65 (0.35–1.23)	0.185	0.79 (0.27–2.38)
≥ 26	2430	13 (535)	0.53 (0.27–1.04)	0.063	0.23 (0.05–1.01)
Smoking history					
no	8069	61 (756)	1		–
yes	1028	10 (973)	1.29 (0.66–2.53)	0.458	–
With in-patient wards					
no	1104	5 (453)	1		–
yes	8232	66 (802)	1.77 (0.71–4.42)	0.216	–
Screening for patients					
yes	6839	49 (716)	1		–
no	2478	22 (888)	1.24 (0.75–2.06)	0.402	–
Items of department and staff composition					
0–1	1819	12 (660)	1		–
2	7517	59 (785)	1.19 (0.64–2.22)	0.582	–
Items of staff training Implementation					
0–2	1269	11 (867)	1		–
3	8067	60 (744)	0.86 (0.45-1.63)	0.639	–
Items of infection control evaluation implementation					
0–1	3164	22 (695)	1		–
2	6172	49 (794)	1.14 (0.69–1.89)	0.604	–
Items of infection control policies and budgets implementation					
2–3	7748	63 (813)	1		1
0–1	1588	8 (504)	0.62 (0.30–1.29)	0.200	0.66 (0.25–1.77)
Items of Individual protection					
6	1872	21 (1122)	1		1
0–5	7464	50 (670)	0.59 (0.36–0.99)	0.047	0.56 (0.29–1.11)
Items of special administrative implementation					
10–12	3779	17 (450)	1		1
0–9	5557	54 (972)	2.17 (1.26–3.75)	0.005	2.57 (1.37–4.80)
Items of promotion and education implementation					
4–6	7087	59 (833)	1		1
0–3	2249	12 (534)	0.64 (0.34–1.19)	0.158	0.64 (0.25–1.61)
Items of floor plan design					
3–4	5694	52 (913)	1		1
0–2	3642	19 (522)	0.57 (0.34–0.96)	0.036	0.68 (0.34–1.36)
Items of ventilation					
2–4	6626	43 (649)	1		1
0–1	2710	28 (1033)	1.60 (0.99–2.58)	0.055	2.42 (1.31–4.47)
Items of ultraviolet germicidal lamps installation and maintenance					
5–6	4455	42 (943)	1		1
0–4	4881	29 (594)	0.63 (0.39–1.01)	0.055	0.81 (0.43–1.55)

The multivariate regression analysis showed that HCWs aged 51 years and above were more susceptible to TB (a*OR* = 6.17, 95% *CI*: 1.35–28.28) and nurses were considered a very high-risk group (a*OR* = 3.09, 95% *CI*: 1.15–8.32). Implementation of 0–9 items of administrative measures (a*OR* = 2.57, 95% *CI*: 1.37–4.80) and of 0–1 items of ventilation measures (a*OR* = 2.42, 95% *CI*: 1.31–4.47) were shown to be risk factors for TB (see Table 6).

## Discussion

This study found that HCWs have a high risk of contracting TB infection due to frequent contact with TB patients and poor observance of protection measures [12, 14, 15]. Although previous reports have described TB prevalence among HCWs in local areas of China [16], this is the first national large sampling survey on TB prevalence in HCWs and associated risk factors, involving both demographics of HCWs and TB infection control policies [16, 17].

The TB prevalence among HCWs in this study was 760/100 000, which is much higher than the TB prevalence in both the general population of China in 2010 (459/100 000) [5] and TB prevalence among HCWs in other countries in 2010 and 2005 respectively [12, 18]. For example, TB prevalence in a survey of 22 healthcare institutions in Beijing, Inner Mongolia, and Shanghai was shown to be 665/100 000 [12]; another survey conducted among HCWs in Mexico showed a prevalence of 439/100 000 [18].

The prevalence of TB among HCWs in eastern China (612/100 000) was lower than that in the west (889/100 000) and central China (919/100 000); both of the latter are considered developing areas when compared to the east. The prevalence in the three areas was also higher than that in the general population (east area: 291/100 000; central area: 463/100 000; west area: 695/100 000) [5]. Other studies showed that the prevalence of LTBIs among HCWs in high-income countries was generally low [19, 20], and a high prevalence of TB was associated with living in poor economic areas and having a low-level education. We did not analyze the association between TB prevalence and economy due to a lack of economic information. However, we did confirm that the prevalence of TB among HCWs in provincial facilities (505/100 000) was lower than that in prefectural (825/100 000) and county-level (836/100 000) facilities. The reason for this might be that provincial facilities had the best practices of TB infection control compared with the prefectural and county-level facilities, which resulted from sufficient manpower and material resources. In addition, HCWs with higher education who had a better understanding of TB infection control practices might pay more attention to TB infection control at higher complexity level facilities.

Nurses were likely to acquire TB in our study, corroborating existing literature (e.g. a study conducted in the Limpopo province of South Africa) [21]. Several previous studies showed a higher TB prevalence of male HCWs than female ones [12, 14, 15, 22], which was contrary to our finding. Our study did not find that HCWs who had worked longer periods in TB facilities were more susceptible to be infected with TB [6, 18, 23]. However, results indicated that HCWs aged 51 years or above were at higher risk of contracting TB, in line with the available literature [24]. This might be due to impaired immunity in older people [25] and higher exposure to TB patients [16]. In terms of occupation, TB prevalence was highest in nurses, followed by doctors. This is probably due to this group’s close contact with patients, particularly the most infectious ones who are subjected to high-risk investigations, such as bronchoscopy examinations [26].

Tuberculosis prevalence among HCWs was associated with administrative control implementation of TB infection control measures, environmental control measures, personal protection [27]. China published a manual for TB infection prevention and control in 2012, which included guidelines primarily on organizational management, plus three other overarching groups of measures: (i) administrative control; (ii) environmental control; and (iii) individual protection [13]. However, these measures were not being implemented well in healthcare facilities [11, 28]. A study in Brazil showed that TB prevalence among HCWs decreased from 580/100 000 to 370/100 000 person-months after implementation of effective administrative control measures [29]. Another study in Northern Ireland showed that TB prevalence among HCWs working in facilities with good implementation of infection control measures and individual protection was not higher than in the general population [30]. Regular screening of HCWs has also been shown to be effective for TB prevention [31].

In this study, the implementation of administrative measures and appropriate ventilation was associated with TB prevalence among HCWs. Lack of good practice in terms of implementation of administrative measures (e.g. independent waiting areas, early diagnosis, cohorts of TB and non-TB patients in wards and outpatient departments) and the absence of appropriate ventilation systems (e.g. removal of mechanical ventilation equipment in outpatient departments or wards) increased the risk of TB.

Our study had limitations that may have impacted on the general validity of the findings. Firstly, facilities were not randomly selected—purposive and typical sampling can cause selection bias. Secondly, data on HCWs were collected by provincial staff members using either personal files or interviews, and false or outdated information may have been collected. Thirdly, HCWs could have been infected either in TB facilities or in the community, and the fact this distinction was not established may have overestimated the prevalence of infection as a result of occupational exposure. Fourthly, not all TB-infected HCWs had microbiological confirmation and this could have also led to overestimation of prevalence. Finally, the cross-sectional design of our study was not ideal to infer causality.

## Conclusions

This was the first large national survey conducted on TB prevalence among HCWs in China. We confirmed that TB prevalence among HCWs is higher than in the general population and is associated with age, professional category, and implementation of managerial infection control measures and ventilation systems in TB facilities. Our study indicates a pressing need to strengthen TB infection control practices in China.

## Additional file

Additional file 1: Multilingual abstract in the five official working languages of the United Nations. (PDF 433 kb)

## Abbreviations

China CDC: Chinese Center for Disease Control and Prevention
*CI*: Confidence interval
HCW: Healthcare worker
LTBI: Latent tuberculosis infection
MTB: *Mycobacterium tuberculosis*
*OR*: Odds ratio
TB: Tuberculosis
