# Supplementary material for: Prevalence of and risk factors for tuberculosis among healthcare workers in Chinese tuberculosis facilities
Source: Infect Dis Poverty. 2018 Mar 24;7:26. doi: 10.1186/s40249-018-0407-6 (PMC5872547; doi:10.1186/s40249-018-0407-6)
Supplement: Supplementary file 1 — Multilingual abstract in the five official working languages of the United Nations. (PDF 433 kb) [file 40249_2018_407_MOESM1_ESM.pdf]

Translation of the abstract into the five official working languages of the United Nations

مدى انتشار عدوى السُّل وعوامل الخطورة بين عاملي القطاع الصحي في المراكز الصينية لعلاج السّل

ملخص:

خلفية: تعد دولة الصين واحدة من22 دولة ذات نسب عالية من حالات السّل في العالم. والعاملون في القطاع الصحي معرضون بدرجة عالية من خطورة الإصابة بعدوى السّل بسبب عدم كفاية الممارسات المستخدمة لمكافحة العدوى. أجرينا دراسة مقطعية لبحث مدى انتشار مرض السل إضافة لعوامل الخطورة المرتبطة به ضمن عاملي القطاع الصحي في مراكز علاج السل في دولة الصين.

منهج الدراسة: أختير 241 مركزا لعلاج السّل من 12 مقاطعة في دولة الصين، بلغ مجموع العاملين فيها 9663 من عاملي القطاع الصحي، لتمثل بيئة المراكز الصحية على مستويات المقاطعة والولاية والدولة. واستخدمت الدراسة استبانات منظمة لجمع بيانات متعلقة بالممارسات المستخدمة لمكافحة السّل وبعاملي القطاع الصحي في تلك المنشآت. أدخلت البيانات إلى برنامج أبيداتا 3،1 Epidata 3.1: وتم تحليل مدى الانتشار وعوامل الخطورة باستخدام برنامج SPSS 21.0 بالاستعانة بنماذج للانحدار الخطي ثنائي المتغيرات ومتعدد المتغيرات

النتائج: أظهرت النتائج تشخيص 71 عاملا في القطاع الصحي بعدوى السّل مما ينم عن مدى انتشار يعادل 760/ 100000. كما أظهر التحليل متعدد المتغيرات أن عوامل الخطورة تشمل الفئة العمرية من 51 عاما فما فوق (a*OR* = 6.17, 95% *CI* = 1.35 – 28.28)، والانتماء لمهنة التمريض (a*OR* = 3.09, 95% *CI* = 1.15 – 8.32)، وتطبيق بين 0-9 إجراءات (a*OR* = 2.57, 95% *CI* = 1.37 – 4.80)، وتطبيق 0-1 إجراءات تهوية (a*OR* = 2.42, 95% *CI* = 1.31 – 4.47).

الاستنتاج: هذه أول دراسة ذات عينة كبيرة وعلى المستوى الدولي في الصين لدراسة مدى انتشار عدوى السّل بين عاملي القطاع الصحي. وقد وجدت ضعف تطبيق ممارسات مكافحة عدوى السّل في بعض المنشآت. نسبة انتشار السّل بين عاملي القطاع الصحي كانت أكبر من النسبة المتواجدة في الجمهور العام. ولذلك يجب تقوية ممارسات مكافحة عدوى السّل في المنشآت الطبية الصينية.

Translated from English version into Arabic by Suzan Alkhodair, through

TRANSLATORS
WITHOUT BORDERS

我国结核病防治机构中医疗卫生工作者的结核患病率及其危险因素的研究

王晓宁，何天伦，耿梦杰，宋渝丹，王吉春，刘敏，Sarah Jayne Hoosdally，Ana Luíza Gibertoni Cruz，赵飞，逄宇，赵雁林，何广学

摘要

引言：中国是世界上 22 个结核病高负担国家之一。由于医疗机构感染控制实施不完全到位，医务人员有感染结核分枝杆菌的风险。我们进行了一项横断面的研究来探索我国结核病防治机构中医疗卫生工作者结核病患病率及其相关危险因素。

方法：在我国 12 省选择省级、市级和县级结核病防治机构共 241 家及其 9663 名医疗卫生工作者作为研究对象，收集医疗机构的结核感染控制情况和医疗卫生工作者信息。使用 EpiData3.1 进行数据双录入；使用 SPSS21.0 对医疗卫生工作者结核患病率及其相关危险因素进行单因素和多因素回归分析。

结果：研究结果显示，被调查人员中，有 71 名医疗卫生工作者患有结核病，患病率为 760/100 000。多因素分析显示，相关的危险因素包括工作人员年龄为 51 岁及以上年龄组(a*OR* = 6.17,

95% *CI* = 1.35 – 28.28)，护士(a*OR* = 3.09, 95% CI = 1.15 – 8.32)，实施管理措施 0-9 项(a*OR* = 2.57, 95% *CI* = 1.37 – 4.80)和通风措施 0-1 项(a*OR* = 2.42, 95% *CI* = 1.31 – 4.47)。
结论：这是在我国第一次大规模关于医疗卫生工作者结核病患病率的调查。研究发现，在部分医疗机构内，结核感染控制实施相对薄弱。医疗卫生工作者结核病患病率比普通人群高。因此，我国的医疗机构中需要强化行结核感染控制措施。

Translated from English version into Chinese by Xiao-Ning Wang

Prévalence et facteurs de risque de tuberculose parmi les travailleurs de santé dans les services chinois de turbeculose

Xiao-Ning Wang1,2†, Tian-Lun He3†, Meng-Jie Geng1†, Yu-dan Song1†, Ji-Chun Wang1†, Min Liu1,4, Sarah Jayne Hoosdally5, Ana Luíza Gibertoni Cruz5, Fei Zhao6*, Yu Pang6*, Yan-Lin Zhao6*, Guang-Xue He1*

Résumé
Contexte: La Chine est l'un des 22 pays avec un fort taux de tuberculose (TB) au monde
Les travailleurs de santé (HCW) ont un haut risque de contracter l'infection Mycobacterium tuberculosis à cause du peu de pratiques de contrôle des infections.
Nous avons mené une étude transversale afin d'étudier la prévalence de TB et ses facteurs de risque associés parmi les HCW dans les installations de tuberculose chinoises.
Méthodes: 241 installations TB employant un total de 9663 HCW ont été controllées dans 12 provinces chinoises afin de représenter les installations de santé au niveau provincial, préfectoral, et national.Des questionnaires structurés ont été donnés pour collecter les informations sur les pratiques de contrôle de l'infection TB et les travailleurs de santé de ces installations.Les données ont été enregistrées deux fois dans EpiData 3.1: la prévalence TB et les facteurs de risque associés ont été analysés en utilisant SPSS 21.0 avec des modèles de régression bivariés et multivariés.
Résultats: Les résultats ont montré que 71 HCW ont été diagnostiqués avec TB, comptabilisant une prévalence de 760 / 100000.L'analyse multivariée a montré que les facteurs de risque associés incluaient: avoir 51 a,s ou plus (aOR = 6.17, 95% CI = 1.35 – 28.28), être une infirmière (aOR = 3.09, 95% CI = 1.15 – 8.32), mise en œuvre de 0 à 9 points de mesures de gestion (aOR = 2.57, 95% CI = 1.37 – 4.80), et mise en œuvre de 0 à 1 point de mesure de ventilation (aOR = 2.42, 95% CI = 1.31 – 4.47).
Conclusions: Ce fut le premier échantillon de questionnaire national sur la prévalence de TB parmi les HCW en Chine.Il a révélé que la mise en œuvre de pratiques de contrôles de l'infection TB dans quelques installations était insuffisante.La prévalence de TB parmi les HCW était plus haute que dans la population en général.Ainsi, les pratiques de contrôle de l'infection TB dans les installations médicales en Chine doivent être renforcées.

Translated from English version into French by Emilie Rigault Fourcadier, through

Распространенность и факторы риска туберкулеза среди работников здравоохранения в китайских противотуберкулезных учреждениях

Xiao-Ning Wang[1,2†], Tian-Lun He[3†], Meng-Jie Geng[1†], Yu-dan Song[1†], Ji-Chun Wang[1†], Min Liu[1,4], Sarah Jayne Hoosdally[5], Ana Lu íza Gibertoni Cruz[5], Fei Zhao[6*], Yu Pang[6*], Yan-Lin Zhao[6*], Guang-Xue He[1*]

Сяо-Нин Ван, Тянь-Лунь Хэ, Мэн-Цзе Гэн, Юй-дань Сун, Цзи-Чунь Ван, Минь Лю, Сара Джейн Хоосдалли, Ана Луиза Гибертони Круз, Фэй Чжао, Юй Пан, Янь-Линь Чжао, Гуан-Сюэ Хэ

Аннотация

История вопроса: Китай является одной из 22 стран с высокой распространенностью туберкулеза в мире. Медицинские работники имеют высокий риск заражения инфекцией микобактерии туберкулеза (*Mycobacterium tuberculosis*) из-за недостаточной практики инфекционного контроля. Мы провели кросс-секционное исследование для изучения распространенности туберкулеза и связанных с ним факторов риска среди медработников в китайских противотуберкулезных учреждениях.

Методы: Двести сорок одно противотуберкулезное учреждение, в которых задействовано всего 9 663 медицинских работников, было отобрано из 12 провинций Китая, чтобы представить медицинские учреждения на уровне провинций, префектур и уездов. Структурированные анкеты были использованы для сбора информации о практике контроля над инфекцией туберкулеза и медицинских работниках в этих учреждениях. Данные были дважды введены в EpiData 3.1; распространенность туберкулеза и связанные с ним факторы риска были проанализированы с использованием SPSS 21.0 с двумерными и многомерными регрессионными моделями.

Результаты: Результаты показали, что туберкулез был обнаружен у 71 медработников с учетом распространенности 760/100 000. Многомерный анализ показал, что связанные факторы риска включали принадлежность к возрастной группе от 51 года и выше (сОШ = 6,17, 95% ДИ = 1,35 - 28,28), работу медсестрой (сОШ = 3,09, 95% ДИ = 1,15 - 8,32), реализацию 0-9 единиц управленческих мер (сОШ = 2,57, 95% ДИ = 1,37 - 4,80) и реализацию 0 - 1 единицы вентиляционных мер (сОШ = 2,42, 95% ДИ = 1,31-4,47).

Выводы: Это обследование было первым национальным крупным выборочным обследованием распространенности туберкулеза среди медработников в Китае. Было обнаружено, что внедрение методов контроля над инфекцией туберкулеза в некоторых учреждениях является неудовлетворительным. Распространенность туберкулеза среди медицинских работников была выше, чем среди общей совокупности населения. Ввиду этого следует усилить практику контроля над инфекцией туберкулеза в китайских медицинских

учреждениях.

Translated from English version into Russian by IraKulinevych, through

TRANSLATORS WITHOUT BORDERS

La prevalencia y los factores de riesgo de tuberculosis entre los profesionales sanitarios en las instalaciones de tuberculosis de China

Xiao-Ning Wang[1,2†], Tian-Lun He[3†], Meng-Jie Geng[1†], Yu-dan Song[1†], Ji-Chun Wang[1†], Min Liu[1,4], Sarah Jayne Hoosdally[5], Ana Luíza Gibertoni Cruz[5], Fei Zhao[6*], Yu Pang[6*], Yan-Lin Zhao[6*], Guang-Xue He[1*]

Resumen

Antecedentes: China es uno de los 22 países con una alta incidencia de tuberculosis en el mundo.Los profesionales sanitarios corren un alto riesgo de contraer la infección de Mycobacterium tuberculosis debido a prácticas de control de la infección insuficientes. Llevamos a cabo un estudio transversal para explorar la prevalencia de la tuberculosis y sus factores de riesgo asociados entre los profesionales sanitarios en las instalaciones de tuberculosis en China.

Métodos: Se seleccionaron doscientas cuarenta y un instalaciones de tuberculosis que empleaban a un total de 9.663 profesionales sanitarios de 12 provincias en China para representar entornos de atención médica a nivel provincial, prefectural y de condado.Los cuestionarios estructurados se utilizaron para recopilar información sobre las prácticas de control de la infección de tuberculosis y sobre los profesionales sanitarios en esas instalaciones.Los datos se ingresaron dos veces en EpiData 3.1; la prevalencia de tuberculosis y los factores de riesgo asociados se analizaron mediante SPSS 21.0 con modelos de regresión bivariantes y multivariados.

Resultados: Los resultados mostraron que 71 profesionales de la salud habían sido diagnosticados con tuberculosis, lo que representa una prevalencia de 760/100 000.El análisis multivariado mostró que los factores de riesgo asociados incluían los pertenecientes al grupo de 51 años de edad o más (aOR = 6.17, 95% CI = 1.35 - 28.28), siendo una enfermera (aOR = 3.09, 95% CI = 1.15 - 8.32), la implementación de 0 - 9 ítems de medidas de gestión (aOR = 2.57, 95% CI = 1.37 - 4.80), y la implementación de 0 - 1 elementos de medidas de ventilación (aOR= 2.42, 95% CI = 1.31 - 4.47).

Conclusiones: Esta fue la primera encuesta nacional amplia por muestreo sobre la prevalencia de tuberculosis entre los profesionles de la salud en China.

Se ha llegado a la conclusión de que la implementación de prácticas de control de infección de tuberculosis en algunas instalaciones era pobre.La prevalencia de la tuberculosis en los profesionales sanitarios fue mayor que en la población general.Por lo tanto, las prácticas de control de infección de tuberculosis en las instalaciones médicas chinas deberían fortalecerse.

Translated from English version into Spanish by nsp, through
